# Absence of SARS-CoV-2 Antibodies in Natural Environment Exposure in Sheep in Close Contact with Humans

**DOI:** 10.3390/ani11071984

**Published:** 2021-07-02

**Authors:** Sergio Villanueva-Saz, Jacobo Giner, Antonio Fernández, Delia Lacasta, Aurora Ortín, Juan José Ramos, Luis Miguel Ferrer, Marta Ruiz de Arcaute, Ana Pilar Tobajas, María Dolores Pérez, Maite Verde, Diana Marteles, Ramón Hurtado-Guerrero, Julián Pardo, Llipsy Santiago, Andrés Manuel González-Ramírez, Javier Macías-León, Ana García-García, Víctor Taleb, Erandi Lira-Navarrete, José Ramón Paño-Pardo, Héctor Ruíz

**Affiliations:** 1 Department of Animal Pathology, Veterinary Faculty, University of Zaragoza, 50013 Zaragoza, Spain; jacoboginer@gmail.com (J.G.); afmedica@unizar.es (A.F.); dlacasta@unizar.es (D.L.); aortin@unizar.es (A.O.); jjramos@unizar.es (J.J.R.); lmferrer@unizar.es (L.M.F.); maltas8@hotmail.com (M.R.d.A.); mverde@unizar.es (M.V.); diana.martelesaragues@gmail.com (D.M.); 2 Clinical Immunology Laboratory, Veterinary Faculty, University of Zaragoza, 50013 Zaragoza, Spain; 3 Instituto Agroalimentario de Aragón-IA2, Universidad de Zaragoza-CITA, 50013 Zaragoza, Spain; atobajas@unizar.es (A.P.T.); dperez@unizar.es (M.D.P.); 4 Department of Animal Production and Sciences of the Food, Veterinary Faculty, University of Zaragoza, 50013 Zaragoza, Spain; 5 Institute for Biocomputation and Physics of Complex Systems (BIFI), Mariano Esquillor s/n, Campus Rio Ebro, Edificio I+D, University of Zaragoza, 50013 Zaragoza, Spain; rhurtado@bifi.es (R.H.-G.); amgonram@gmail.com (A.M.G.-R.); jvmacleo@gmail.com (J.M.-L.); anaauxi.garcia@gmail.com (A.G.-G.); v.taleb.mail@gmail.com (V.T.); erandi@bifi.es (E.L.-N.); 6 Aragon I+D Foundation (ARAID), 50018 Zaragoza, Spain; pardojim@unizar.es; 7 Laboratorio de Microscopías Avanzada (LMA), Mariano Esquillor s/n, Campus Rio Ebro, Edificio I+D, Copenhagen Center for Glycomics, 50018 Zaragoza, Spain; 8 Copenhagen Center for Glycomics, Department of Cellular and Molecular Medicine, University of Copenhagen, 2200 Copenhagen, Denmark; 9 Aragon Health Research Institute (IIS Aragón), 50009 Zaragoza, Spain; llipsysg@gmail.com; 10 Department of Microbiology, Pediatrics, Radiology and Public Health, Zaragoza University of Zaragoza, 50013 Zaragoza, Spain; 11 Infectious Disease Department, University Hospital Lozano Blesa, 50009 Zaragoza, Spain; joserrapa@gmail.com

**Keywords:** coronavirus disease 2019 (COVID-19), ELISA, SARS-CoV-2, serology, sheep, ruminants

## Abstract

**Simple Summary:**

Different species can harbor coronavirus, including domestic and wild animals. The Coroviridae family is composed of four genera, including *Alphacoronavirus, Betacoronavirus, Gammacoronavirus* and *Deltacoronavirus*. Some domestic animals are susceptible to SARS-CoV-2 in natural and experimental infections. The infection of animals is generally a consequence of close contact with COVID-19 patients. Among domestic animals, SARS-CoV-2 replicates in respiratory ex vivo organ cultures of domestic ruminants. In this context, there is no information about the role sheep can play in the spread of the infection. This study tested the antibody response in 90 serum samples from sheep from the pre-pandemic period and 336 serum samples from sheep from the pandemic period (June 2020 to March 2021). In both cases, the animals were in close contact with a veterinary student community composed of more than 700 members. None of the serum samples analyzed were seroreactive based on an enzyme-linked immunosorbent assay (ELISA) using the receptor-binding domain (RBD) of the spike antigen. In this sense, no statistical difference was observed compared to the pre-pandemic sheep. To the authors’ knowledge, this is the first serosurvey in sheep to detect anti-SARS-CoV-2 antibodies. Future investigations should analyze the epidemiological role of sheep in SARS-CoV-2 infection, including the prevalence of infection.

**Abstract:**

Severe acute respiratory syndrome coronavirus 2 (SARS-CoV-2) is the zoonotic causative agent of coronavirus disease 2019 (COVID-19) that has caused a pandemic situation with millions of infected humans worldwide. Among domestic animals, there have been limited studies regarding the transmissibility and exposure to the infection in natural conditions. Some animals are exposed and/or susceptible to SARS-CoV-2 infection, such as cats, ferrets and dogs. By contrast, there is no information about the susceptibility of ruminants to SARS-CoV-2. This study tested the antibody response in 90 ovine pre-pandemic serum samples and 336 sheep serum samples from the pandemic period (June 2020 to March 2021). In both cases, the animals were in close contact with a veterinary student community composed of more than 700 members. None of the serum samples analyzed was seroreactive based on an enzyme-linked immunosorbent assay (ELISA) using the receptor-binding domain (RBD) of the spike antigen. In this sense, no statistical difference was observed compared to the pre-pandemic sheep. Our results suggest that it seems unlikely that sheep could play a relevant role in the epidemiology of SARS-CoV-2 infection. This is the first study to report the absence of evidence of sheep exposure to SARS-CoV-2 in natural conditions.

## 1. Introduction

Severe acute respiratory syndrome coronavirus 2 (SARS-CoV-2) is the zoonotic causative agent of the coronavirus disease 2019 (COVID-19) pandemic which has infected millions of humans worldwide. This is a novel coronavirus that emerged towards the end of 2019. However, different species can harbor coronavirus, including domestic and wild animals. The Coroviridae family is composed of four genera, including *Alphacoronavirus, Betacoronavirus, Gammacoronavirus* and *Deltacoronavirus*. Numerous domestic and pet animals that regularly come into close contact with humans may be susceptible to SARS-CoV-2 infection. Animal infection associated with SARS-CoV-2 has been described in both natural conditions and experimental infections, including dogs, ferrets, cats, lions and minks, among others [1]. Moreover, exposure to SARS-CoV-2 in the absence of clinical signs has been recently described in Spain in stray cats [2] and household ferrets [3].

A different way to assess the susceptibility of animals to the infection is based on alternative procedures, such as in silico and in vitro analyses. Studies evaluating the structural interplay between the receptor-binding domain (RBD) of the SARS-CoV-2 spike protein (S-protein) and the extracellular peptidase domain of angiotensin I converting enzyme 2 (ACE2) show that among agricultural species, ruminants have comparable levels of binding S-protein:ACE2 affinities to humans [4]. Therefore, sheep that come into close contact with humans could be at risk of infection by SARS-CoV-2. There is strong evidence that SARS-CoV-2 presents respiratory tropism in ruminant tissues, including in cattle and sheep. This virus is able to replicate in respiratory ex vivo organ cultures, and the virus is associated with ACE2-expressing cells of the respiratory tract of both ruminant species [5].

In natural conditions, the role of sheep in spreading infection in the community is unknown. Several studies have provided the seroprevalence and molecular data of different animals, but considerable gaps remain regarding ruminants, particularly sheep. The aim of the present study was to determine the exposure to SARS-CoV-2 by detecting anti-SARS-CoV-2 antibodies using an ELISA method. For this, samples of sheep were taken from the farm of a university campus of the Veterinary Faculty, where the sheep are in close contact with many students who carry out their experiments on them.

## 2. Materials and Methods

### 2.1. Study Area, Sampling and Data Collection

Three hundred and thirty-six residual sheep (Rasa Aragonesa breed) serum samples were obtained from 285 animals at the Veterinary Faculty of Zaragoza (41°39′24.6276″ N, 0°52′45.912″ W). Based on an expected seroprevalence range of 3.3 to 5.8% [6], an accepted deviation of the true prevalence of 5% and a confidence level of 95% of the sample size was necessary to estimate the seroprevalence calculated, including a minimum of 50 (3.3% seroprevalence) samples and a maximum of 84 samples (5.8% seroprevalence). In total, 285 animals were sampled, including 35 healthy animals belonging to the productive flock of the university farm and 250 clinical cases referred by veterinarians from the Faculty’s area of influence. Serum samples were collected aseptically by jugular vein venipuncture associated with routine healthcare check-ups from June 2020 to March 2021. The separated residual sera were stored at −20 °C until they were processed.

In 51 of the sheep analyzed, the sample was taken at two different timepoints: upon arrival at the service in June/July 2020 and during March 2021 in order to detect a possible seroconversion. In the rest of the sheep (234 animals), only one single sample was analyzed during the pandemic period.

It is essential to highlight that these animals belong to the university farm. In the Ruminant Clinical Service, a small healthy productive flock is maintained, and many referred cases are received from different farms in the northwestern area of Spain. These sheep are kept in our facilities for an average period of 3 to 4 months. All the sheep are used for routine veterinary degree practices. During the whole academic year, there are more than 650 students who receive teaching at the university sheep farm, as well as teachers and staff involved in the care and maintenance of the animals. It is known that some of the people in charge of the care of the sheep suffered from COVID-19 during the analyzed period.

### 2.2. Expression and Purification of the Receptor Binding Domain (RBD) of the Spike Protein

The DNA sequence encoding amino acid residues 319-541 (RVQPTESIVRFPNITNLCPFGEVFNATRFASVYAWNRKRISNCVADYSVLYNSASFSTFKCYGVSPTKLNDLCFTNVYADSFVIRGDEVRQIAPGQTGKIADYNYKLPDDFTGCVIAWNSNNLDSKVGGNYNYLYRLFRKSNLKPFERDISTEIYQAGSTPCNGVEGFNCYFPLQSYGFQPTNGVGYQPYRVVVLSFELLHAPATVCGPKKSTNLVKNKCVNF) of the RBD was codon optimized and synthesized by GenScript (USA) for expression in HEK293 cells. The DNA, containing at the 5′-end a recognition sequence for KpnI, and at the 3′-end a stop codon and a recognition sequence for XhoI, was cloned into a modified pHLSec containing a 12xHis tag, a superfolder GFP and a tobacco etch virus (TEV) cleavage site after the secretion signal sequence, rendering the vector pHLSec-12His-GFP-TEV-SRBD. Both the synthesis of the RBD construct and the engineered pHLSec together with the cloning of RBD into pHLSec-12His-GFP-TEV were performed by GenScript. The vector pHLSec-12His-GFP-TEV-RBD was transfected into the HEK293F cell line (Thermo Fisher Scientific) as described below. Cells were grown in suspension in a humidified 37 °C and 8% CO_2_ incubator with a rotation of 125 r.p.m. Transfection was performed at a cell density of 2.5 × 106 cell/mL in fresh F17 serum-free media with 2% Glutamax and 0.1% P188. For each 150 mL of culture, 450 μg of the plasmid (1μg/μL) was diluted to 135 μL with sterilized 1.5 M NaCl. This mixture was added to each 150 mL cell culture flask and incubated for 5 min in the incubator. After that, 1.35 mg of PEI-MAX (1 mg/mL) was made up to 135 μL with sterilized 1.5 M NaCl and added to the cell culture flask. Cells were diluted 1:1 with pre-warmed media supplemented with valproic acid 24 h post-transfection to a final concentration of 2.2 mM. Cells were harvested 6 days post-transfection by spinning down at 300× *g* for 5 min, after which the supernatants were collected and centrifuged at 4000× *g* for 15 min. The supernatant was dialyzed against buffer A (25 mM TRIS pH 7.5, 300 mM NaCl) and loaded into a His-Trap column (GE Healthcare). Protein was eluted with an imidazole gradient in buffer A from 10 mM up to 500 mM. Buffer exchange to 25 mM TRIS pH 7.5, 150 mM NaCl (buffer B) was carried out using a HiPrep 26/10 desalting column (GE Healthcare). TEV protease was then added in a ratio of 1:50 (TEV:RBD) to the fusion construct in order to cleave the His-GFP. After 20 h of reaction at 18 °C, the cleavage was satisfactorily verified through SDS-PAGE. TEV protease and GFP were removed from the solution using a His-Trap column (GE Healthcare), and the SRBD was collected from the flow-through. Quantification of protein was carried out by absorbance at 280 nm using the theoretical extinction coefficient, ε280 nm (RBD) = 33,350 M^−1^cm^−1^.

### 2.3. Detection of SARS-CoV-2 Antibodies by In-House ELISA

An in-house indirect ELISA was established for the detection of IgG specific for RBD of the spike protein [2,3]. Ninety-six-well plates were coated overnight, at 4 °C with 100 ng RBD protein in phosphate-buffered saline (PBS). Subsequently, the coating solution was removed, and the plate was washed three times with 200 μL per well of PBS + TWEEN 0.05% (PBST). Three hundred microliters of PBST and 3% dry skimmed milk was added to each well as blocking solution. The plate was incubated with blocking solution for 1 h at 37 °C in a moist chamber. One hundred microliters of ferret sera, diluted 1:100 in PBS containing 0.05% Tween 20 and 1% dry skimmed milk (PBST-M), was added to each well. The plates were incubated for 1 h at 37 °C in a moist chamber. After washing the plates for 30 s 6 times with PBST followed by one wash with PBS for 1 min, 100 µL/well of multi-species horseradish peroxidase conjugate (Thermo Fisher Scientific, Waltham, MA, USA), diluted 1:100,000 in PBST-M, was added per well. The plates were incubated for 1 h at 37 °C in the moist chamber and were washed again with PBST and PBS as described above. The substrate solution (ortho-phenylene-diamine) and stable peroxide substrate buffer (Thermo Fisher Scientific, Waltham, MA, USA) were added at 100 µL per well and developed for 20 ± 5 min at room temperature in the dark. The reaction was stopped by adding 100 µL of 2.5 M H_2_SO_4_ to each well. Absorbance values were read at 492 nm in an automatic microELISA reader (ELISA Reader Labsystems Multiskan, Midland, ON, Canada). As a positive control, each plate included two serum samples, including a human patient diagnosed with COVID-19, confirmed by a molecular test and a commercial quantitative ELISA, and a serum sample from a seropositive cat for SARS-CoV-2 [3]. By contrast, serum from healthy, non-infected sheep obtained prior to the COVID-19 pandemic was used as a negative control. The same positive and negative sera were used for all assays. All samples were run in duplicate. The cutoff was set to 0.174 optical density units (OD units) (mean + 3 standard deviations of values from 90 sheep obtained prior to the COVID-19 situation between 2017 and 2018), and results above this value were considered positive.

## 3. Results

### 3.1. Characterization of the Analyzed Animals

One hundred and fifty-five out of the 285 analyzed animals were lambs. Ninety-four were lambs aged between 73 and 112 days from a clinical case of ovine anaplasmosis (41 females and 53 males), and sixty-one were 2- to 6-month-old lambs with poor growth rates (29 females and 32 males). The causes of this poor growth rate included locomotive (45/61), digestive (8/61), respiratory (4/61) and neurological disorders (4/61).

One hundred and thirty out of the 285 analyzed animals were adult sheep. Thirty-five were 10-month-old to 9-year-old apparently healthy sheep from the permanent flock of the Veterinary Campus (32 females and 3 males), and 95 were 1- to 10-year-old adult sheep from referred clinical cases (88 females and 7 males). Table 1 shows the type of disorders detected in this group of 95 sheep. Out of the 51 animals sampled twice, 36 belonged to the group of sick adult animals, while the remaining 15 animals were lambs with poor growth rates.

A total of 19 sheep from a farmer with a positive COVID-19 diagnosis in September 2020 were included: six serum samples were obtained in October 2020, five samples in December 2020, and finally eight serum samples in February 2021.

### 3.2. Serological Prevalence of SARS-CoV-2 Infection in Sheep

Anti-SARS-CoV-2 antibodies were not detected in the sheep included in this study, and no differences were observed compared to the OD values from 90 sheep obtained prior to the COVID-19 situation. Therefore, none of the farm animals included in this study had been exposed to SARS-CoV-2, despite repeated daily close contact with veterinary students on the campus from June 2020 to March 2021.

## 4. Discussion

To date, studies investigating SARS-CoV-2 in sheep are scarce; most of the previous studies focus on research of human diseases or on other animals. It seems proven that SARS-CoV-2 has infected domestic [7] and wild animals through contact with infected humans [8]. The main exception is related to minks, with the transmission to humans being possible through close contact with them in high-density mink farms [9]. The absence of exposure in all sheep despite close contact with the humans of this study probably indicates that sheep are not susceptible to SARS-CoV-2 infection.

In order to evaluate animal susceptibility to SARS-CoV-2, predictive analyses of the binding potential of SARS-CoV-2 with the ACE2 receptor could be performed including in vitro and in silico analyses. By contrast, another type of evaluation can be based either on experimental infections of SARS-CoV-2, or field studies based on observations in natural conditions. Among ruminants, cattle are the species in which SARS-CoV-2 susceptibility has been studied most extensively. Two experimental infections have been carried out. In the first, six cattle were infected utilizing an intranasal inoculation [10], and in the second study, intratracheal and intravenous SARS-CoV-2 inoculation in six colostrum-deprived calves was performed [11]. Results obtained from both studies revealed that cattle have a low susceptibility to SARS-CoV-2 infections. However, some inconsistency is shown between in vitro and in vivo results. Replication of SARS-CoV-2 in cattle cells was reported in vitro [5,11], suggesting that ACE2 expression in respiratory tract cells is very variable, and suggesting deeper observations in natural and experimental conditions are necessary.

Likewise, potential susceptibility to SARS-CoV-2 has been studied in white-tailed deer (*Odocoileus virginianus*) [12]. The susceptibility of deer cells to SARS-CoV-2 infection and replication was assessed in in vitro conditions, sharing a high degree of similarity of ACE2 to humans. Moreover, experimental infection was carried out in white-tailed deer, and it was revealed that subclinical infection was possible, together with the presence of the virus in nasal secretions. In this case, in vitro and in vivo analyses are complementary to identify the SARS-CoV-2 host range. If these findings are consistent, it could provide important insight when considering deer as a potential reservoir for SARS-CoV-2.

Animal models have been used in experimental research to increase human knowledge of the epidemiological role of different animal species and contribute to finding solutions to SARS-CoV-2 infection [10]. However, these procedures are not without significant costs, including biocontainment facilities with a biosafety level of 3 and special personnel requirements which are not always possible to obtain in research conditions.

The main difference between field study observation and experimental infection is the type of sample used. The cattle studied in the cited articles were experimentally infected by intratracheal and intravenous inoculation with a high dose of virus, whilst the present study enrolled sheep with potential exposure to SARS-CoV-2 in natural conditions in close contact with humans. The evolution and pathogenesis of natural infection in animals is highly variable and not easily comparable with experimental infection. It should be pointed out that the administration of virus for experimental infection induces a rapid progression of clinical signs and lesions when compared to natural infection. For this reason, it is important to note that there may also be differences between experimental and natural infections in the routes of inoculation and the route of infection.

Observational field studies are focused on monitoring SARS-CoV-2 natural infection, with different approaches such as the detection of active cases of SARS-CoV-2, including the emergence of new variants of SARS-CoV-2 with protein mutations, or indirect diagnosis based on measuring exposure level of animals to SARS-CoV-2 by cross-sectional serological surveys. Different confirmatory laboratory tests available for animal species include a molecular test using reverse transcription polymerase chain reaction (RT-PCR) or a serological test such as ELISA. From a transmission point of view, both approaches are recommended to investigate whether these new variants could be transmitted among animals or are involved with backward transmission [13].

Different types of ELISA are available; some of them are commercial, and others are of an in-house nature. Two commercial ELISAs based on double antıgens (ID Screen^®^ SARS-CoV-2 Double Antigen Multi-species ELISA [14]; WANTAI SARS-CoV-2 Ab ELISA [15]) are available for serological testing in veterinary laboratories. In general, for animal diagnosis an ELISA targeting multiple species is recommended. There are different types of antigen that can be used to sensitize plates, including a whole virus, the major nucleocapsid protein (N antigen), the membrane proteins (M antigen), the spike protein (S antigen) or the RBD antigen of SARS-CoV-2 [16,17,18]. Likewise, we have selected for our study an in-house ELISA coated with the RBD antigen because better diagnostic measures were obtained in comparison to the remaining antigens in terms of specificity. Other techniques to characterize serological responses to SARS-CoV-2 include SARS-CoV-2 titration, micro-neutralization assays [19], Western blotting [20], immunoblotting assay [21], luciferase immuno-precipitation technique [22] or microsphere immunoassay [23]. ELISA has a number of benefits compared to the other immunoassay techniques, including that it is a simple procedure with a 96-well microplate format in which simultaneous analyses can be performed. By contrast, the remaining techniques are limited to research due to having sophisticated techniques, and samples often needing to be sent to a specialized laboratory to perform the assays. Moreover, SARS-CoV-2 titration and micro-neutralization assays are labor-intensive and expensive, requiring trained personnel with specific laboratory requirements such as culture areas and biosafety cabinets. ELISA techniques are widely used in this type of seroepidemiological survey performed in animals to detect the presence of anti-SARS-CoV-2 antibodies [2,3,14,24].

The circumstances that can affect the performance of a serological diagnostic technique include the type of matrix used, such as whole blood, plasma, serum or even filter paper blood samples. These circumstances are important due to the fact that the ID Screen^®^ SARS-CoV-2 Double Antigen Multi-species ELISA recommends samples of serum, plasma or whole blood, whilst the WANTAI SARS-CoV-2 Ab ELISA was developed for plasma or serum samples. Samples with anticoagulants may lead to non-specific reactions, obtaining false-positive results. In general, it is recommended to use serum for serological tests as was done in our study.

The lack of a reliable SARS-CoV-2 gold standard technique in animals, together with the absence of appropriate validation of animal studies, is a common problem observed in the scientific literature. Different approaches could be performed, including using serum samples obtained from infected animals in experimental studies as seropositive samples, or using samples collected before the SARS-CoV-2 period from animal serum biobanks as negative controls [18]. By contrast, the development of multi-species ELISAs has a significant advantage; human samples could be used to perform validation parameters, as is the approach used for the WANTAI SARS-CoV-2 Ab ELISA. In this study, we have employed an ELISA previously developed in our lab based on the RBD human SARS-CoV-2 antigen that has been previously found to be useful in detecting neutralizing anti-SARS-CoV-2 antibodies in different hosts, including humans [19], cats [2], ferrets [3] and experimental mice (unpublished). Thus, the absence of positive results in sheep is unlikely to be due to the poor performance of this ELISA in detecting sheep-derived anti-SARS-CoV-2 antibodies.

Finally, a potential limitation of this study is the lack of data on the number of students infected that were in close contact with the sheep; however, this type of information is confidential and, in general, it is not available.

## 5. Conclusions

This study reports, for the first time, the absence of sheep household exposure to SARS-CoV-2 to date. Our findings suggest the absence of virus exposure between sheep and humans, including the veterinary student community, is likely due to the lower susceptibility of sheep. The existence of concomitant diseases and the lack of anti-SARS-CoV antibodies after exposure do not suggest that immunosuppressed animals might be especially susceptible to SARS-CoV-2 infection, a situation that is different in other species such as cats and ferrets. Further studies are necessary to evaluate the impacts of other potentially susceptible animal species previously detected by in vitro studies.

## Figures and Tables

**Table 1:** Types of disorders detected in the group of 95 sheep.

Type of Disorder	Number of Animals (n = 95)	Clinical Entities
Respiratory disorders	24	Ovine pulmonary adenocarcinoma, small ruminant lentivirosis, ovine respiratory complex, verminous pneumonia, gangrenous pneumonia, enzootic nasal adenocarcinoma and oestrosis
Digestive disorders	11	Paratuberculosis, small intestinal adenocarcinoma, chronic bloat and ruminant sores
Mammary gland disorders	11	cronic mastitis and gangrenous mastitis
Anaplasmosis	8	Ovine anaplasmosis
Locomotive disorders	6	Osteoarthritis, arthritis, fractures and laminitis
Caseous lymphadenitis	5	Visceral presentation
Jaw and maxillary osteomyelitis	5	
Neurological disorders	4	Small ruminant lentivirosis, intoxication and traumatic contusions
Reproductive disorders	3	Metritis
Dermatological disorders	3	Congenital disorders and skin tumors
Internal abscesses	2	Visceral presentation
Heart disorders	1	
Animals without definitive diagnosis	12	

## Data Availability

The data presented in this study are available on request from the corresponding author. The data are not publicly available due to privacy restrictions.
